# Pyrite-induced hydroxyl radical formation and its effect on nucleic acids

**DOI:** 10.1186/1467-4866-7-3

**Published:** 2006-04-04

**Authors:** Corey A Cohn, Steffen Mueller, Eckard Wimmer, Nicole Leifer, Steven Greenbaum, Daniel R Strongin, Martin AA Schoonen

**Affiliations:** 1 Department of Geosciences, Stony Brook University, Stony Brook, NY 11794, USA; 2 Department of Molecular Genetics and Microbiology, Stony Brook University, Stony Brook, NY 11794-2100, USA; 3 Department of Physics, Hunter College, New York, NY 10021, USA; 4 Department of Chemistry, Beury Hall 201, 1901 N. 13th Street, Temple University, Philadelphia, PA 19122, USA; 5 Center for Environmental Molecular Science, Stony Brook University, Stony Brook, Stony Brook, NY 11794-2100, USA

## Abstract

**Background:**

Pyrite, the most abundant metal sulphide on Earth, is known to spontaneously form hydrogen peroxide when exposed to water. In this study the hypothesis that pyrite-induced hydrogen peroxide is transformed to hydroxyl radicals is tested.

**Results:**

Using a combination of electron spin resonance (ESR) spin-trapping techniques and scavenging reactions involving nucleic acids, the formation of hydroxyl radicals in pyrite/aqueous suspensions is demonstrated. The addition of EDTA to pyrite slurries inhibits the hydrogen peroxide-to-hydroxyl radical conversion, but does not inhibit the formation of hydrogen peroxide. Given the stability of EDTA chelation with both ferrous and ferric iron, this suggests that the addition of the EDTA prevents the transformation by chelation of dissolved iron species.

**Conclusion:**

While the exact mechanism or mechanisms of the hydrogen peroxide-to-hydroxyl radical conversion cannot be resolved on the basis of the experiments reported in this study, it is clear that the pyrite surface promotes the reaction. The formation of hydroxyl radicals is significant because they react nearly instantaneously with most organic molecules. This suggests that the presence of pyrite in natural, engineered, or physiological aqueous systems may induce the transformation of a wide range of organic molecules. This finding has implications for the role pyrite may play in aquatic environments and raises the question whether inhalation of pyrite dust contributes to the development of lung diseases.

## Introduction

Earlier studies have shown that pyrite/aqueous suspensions generate hydrogen peroxide (H_2_O_2_) in the absence of oxygen [1] and during pyrite oxidation [2]. While the formation of H_2_O_2 _has been established in pyrite suspensions, its fate is not clear. Borda and coworkers reported a single experiment in which the formation of hydroxyl radical was inferred on the basis of the transformation of a radical scavenger [3]. Cohn and coworkers speculated that the H_2_O_2 _reacts with ferrous iron to form hydroxyl radicals (^•^OH) via a Fenton reaction mechanism [4]. This notion was based on two sets of experimental observations. Firstly, RNA is readily degraded in pyrite/aqueous suspensions [4]. Secondly, RNA is stable in the presence of H_2_O_2 _[5], but degrades rapidly in the presence of ^•^OH [6,7]. The latter observation is also consistent with experimental data correlating particle-induced ^•^OH formation and carcinogenesis and oxidative stress [8,9]. Reactive oxygen species include H_2_O_2 _and the particularly reactive ^•^OH. The extreme reactivity of ^•^OH has been implicated in several diseases [10,11] and the reactivity of ^•^OH toward organics has been exploited in the remediation of organic solvents [12,13].

In this contribution, the hypothesis that pyrite-generated H_2_O_2 _will react with ferrous iron at the pyrite surface or dissolved from pyrite is tested. Evidence for the formation of ^•^OH is presented. This evidence is based on a combination of conventional electron spin resonance spectroscopic (ESR) methods to detect ^•^OH and the kinetics of the degradation of nucleic acids (RNA and DNA) in pyrite/aqueous suspensions. The results of this study may explain the reactivity of pyrite towards other biomolecules, such as purine nucleosides and formate [14]. The spontaneous formation of ^•^OH in pyrite slurries may also be used to develop environmental remediation strategies. Within the last decade, *in situ *organic pollutant oxidation techniques based on a reaction between hydrogen peroxide and iron-containing oxides have been developed [12,15-18] and applied in the field [19]. This finding suggests that pyrite may be an effective alternative to iron oxides in these applications.

## Background

In this section the pathways that can lead to H_2_O_2 _formation in pyrite slurries and the transformation of H_2_O_2 _to ^•^OH via the Fenton reaction are briefly summarized.

### Formation of H_2_O_2_ in pyrite slurries

The presence or absence of molecular oxygen (O_2_) dictates the reaction pathways that are possible for the formation of H_2_O_2_. In the presence of O_2_, the formation of H_2_O_2 _in pyrite slurries may proceed via two non-exclusive pathways. As pyrite is dispersed in water, ferrous iron will readily be released to solution. Dissolved O_2 _reacts with dissolved Fe(II) via the Haber-Weiss reaction mechanism and forms H_2_O_2 _(eqs 1 and 2) with superoxide (O_2_^•^)^- ^as an intermediate species.

Fe^II^(aq) + O_2 _→ Fe^III^(aq) + (O_2_^•^)^- ^    (1)

Fe^II^(aq) + (O_2_^•^)^- ^+ 2H^+ ^→ Fe^III^(aq) + H_2_O_2 _    (2)

This homogeneous pathway is complemented by a very similar reaction in which O_2 _is reduced by surface-bound Fe(II) (eqs 3 and 4). In the system of interest here, surface-bound ferrous iron can be either Fe(II) that is part of the pyrite crystal structure or sorbed onto the pyrite surface.

Fe^II^(pyrite) + O_2 _→ Fe^III^(pyrite) + (O_2_^•^)^- ^    (3)

Fe^II^(pyrite) + (O_2_^•^)^- ^+ 2H^+ ^→ Fe^III^(pyrite) + H_2_O_2 _    (4)

On the pyrite surface this sequence of reactions may be effectively accomplished by a single two-electron transfer reaction involving two Fe(II) atoms. By contrast, the homogeneous reaction (eqs 1 and 2) has to proceed as two separate one-electron transfer reactions because each ferrous iron can only donate one electron. In addition, the ferric iron species formed on pyrite may readily be reduced by the pyrite substrate. This reduction would make it possible to rapidly cycle the iron between the two oxidation states with O_2 _as the terminal electron acceptor. As a result, the reaction on the pyrite surface is expected to be much faster. This notion is supported by experimental results for other electron transfer processes mediated by mineral surfaces [20].

Borda et al. [3] showed that pyrite can also generate H_2_O_2 _in the absence of molecular oxygen. On the basis of experiments with isotopically labelled water, they proposed that the formation is driven by a reaction between water and a sulphur-deficient defect on the pyrite surface. In brief, bulk pyrite is composed of ferrous iron and disulfide. Sulfur in the disulfide moiety has an oxidation state of -I. However, surface science studies show that sulphur-deficient defects are common on pyrite [21-23]. The sulphur associated with these defects is sulphur with a -II oxidation state [24-26]. In order to maintain charge, this requires that some of the iron is nominally present in the +III state. X-ray photoelectron spectroscopy (XPS) results presented by Borda et al [3] show the presence of some Fe(III) on the pyrite surface. These Fe(III) surface species are reduced to Fe(II) species upon exposure to water. In this reaction an electron is extracted from water and a hydroxyl radical is formed. Combining two hydroxyl radicals leads to the formation of H_2_O_2_. Hence, the overall pathway in the absence of oxygen may be represented as:

Fe^III^(pyrite) + H_2_O → Fe^II^(pyrite) + ^•^OH + H^+ ^    (5)

2^•^OH → H_2_O_2 _    (6)

The Fe(III) coordinated by disulfide groups is energetically highly unfavourable, while the pyrite structure with iron in the divalent state is energetically very favourable. The reaction may be promoted by this difference in energy.

### Fenton reaction

The Fenton reaction refers to the transformation of H_2_O_2 _to ^•^OH via a reaction involving ferrous iron (eq. 7). The rapid and quantitative reaction converts H_2_O_2 _to ^•^OH [27]. If ferric iron formed in the reaction is reduced, then the ferrous iron is recycled and it acts as a catalyst. The Fenton reaction is widely used in the biomedical research community to generate ^•^OH. By binding a ferrous iron complex onto a particular section of a nucleic acid sequence it is possible to induce strand scissions at specific locations [28-30]. Generating ^•^OH is often accomplished by mixing a ferrous salt solution, typically ferrous ammonium sulfate, with a dilute hydrogen peroxide solution. If a reductant, such as ascorbic acid is added, the Fe(III) can be recycled back to Fe(II) (eq. 8). The overall process can be represented by the following two reactions:

Fe^II^(aq) + H_2_O_2 _→ ^•^OH + OH^- ^+ Fe^III^(aq)     (7)

Fe^III^(aq) + Reductant → Fe^II^(aq) + Reductant^+ ^    (8)

While the Fenton reaction, as represented by equations 7 and 8, is a homogeneous reaction, the reaction can also proceed when ferrous iron is present as a solid. For example, Watts and coworkers [15] have shown that magnetite facilitates the conversion of H_2_O_2 _to ^•^OH via the Fenton reaction mechanism. Even Fe(III)-oxides and (oxy)hydroxides can facilitate the reaction albeit less effectively than magnetite [15,31]. For Fe(III)-oxide minerals, Fe(III) may be reduced in the presence of H_2_O_2 _through the following reaction:

Fe^III^(oxide mineral) + H_2_O_2 _→ Fe^II^(oxide mineral) + (O_2_^•^)^- ^+ 2H^+ ^    (9)

Hence, in this study we propose that pyrite can promote the Fenton reaction. Given the rapid oxidation and release of iron into solution when pyrite is dispersed in water, pyrite may promote the Fenton reaction by providing dissolved iron or via a surface reaction. Regardless whether the Fenton reaction takes place in solution or on its surface, pyrite can readily reduce ferric iron. The reaction between dissolved ferric iron and pyrite has been studied extensively in the context of acid mine drainage [32,33]. The semiconducting properties of pyrite [34] allow for rapid reduction of ferric iron that is either formed on the surface or that adsorbs onto its surface.

## Research strategy

Demonstrating the presence of radical species, including ^•^OH, in any system is a challenge given the fact that these species are very short-lived. The most commonly used detection technique for mineral-generated ^•^OH is electron spin resonance [ESR, also known as electron paramagnetic resonance (EPR)] using 5, 5-dimethyl, 1-pyrroline *N*-oxide (DMPO) as a *spin-trap*. The lifetime of ^•^OH is on the order of a few nanoseconds, but its reaction through addition with DMPO results in the lone electron being *trapped *on DMPO to form the DMPO-OH radical adduct. The adduct has a much longer lifetime and can be detected. The problem with using ESR spin-trapping with DMPO is its susceptibility to artifacts. In the case of studies involving iron-bearing minerals, it is important to note that the presence of ferric iron can induce the formation of a DMPO-OH adduct in the absence ^•^OH [35]. However, there is a standard protocol to verify the presence of radicals. In this protocol another reactant, which acts as a *radical scavenger *(e.g., ethanol), is added to compete with DMPO for reaction with ^•^OH. When ethanol is used as a radical scavenger, its reaction with ^•^OH forms the α-hydroxyethyl radical that adds to DMPO resulting in the DMPO-CH(CH_3_)OH adduct [35]. In the absence of ^•^OH, but in the presence of ferric iron (or other nucleophiles), ethanol will attack DMPO by addition with its oxygen and not carbon moiety. This then results in the formation of a DMPO-OCH_2_CH_3 _adduct [35]. The DMPO-CH(CH_3_)OH and DMPO-OCH_2_CH_3 _adducts have different ESR spectra. Hence, in this study we conducted an array of spin-trapping experiments to conclusively demonstrate the formation of ^•^OH in pyrite slurries.

While ESR spin-trapping is the standard method in demonstrating the formation of radical species, it is difficult to conduct spin-trap experiments under strictly anaerobic conditions due to the size of the instrument. Furthermore, the technique provides an indication of the type of radical(s) present and approximate concentrations, but the technique does not lend itself easily to compare a suite of minerals or reaction conditions.

In this study, we used several methods that rely on the interaction of ^•^OH with nucleic acids to complement the spin-trapping experiments. Minerals that induce the formation of ^•^OH have been shown to cause strand breakage in DNA [9,36], which is of interest in genotoxicology (for review see [36]). In previous studies, we have employed short-strand yeast RNA as a probe for mineral-generated ^•^OH [37]. ^•^OH preferentially attacks the deoxyribose moieties in DNA or RNA strands. The resulting shortening of the strands can be detected using various techniques, see method section below. In this study, we used several different experimental procedures to detect strand shortening of RNA and/or DNA exposed to pyrite slurries. Experiments with yeast RNA were performed to evaluate whether pyrite's reactivity toward nucleic acids is limited to a single burst upon exposure to an aqueous solution. Yeast RNA was exposed to pyrite and after more than 98% of the RNA was undetected, a new solution of RNA was added to the pyrite. The experiments with ribosomal RNA and DNA were performed to evaluate their stabilities in the presence of pyrite as a function of time and as a function of total surface area of pyrite, respectively.

Control experiments with RNA exposed to dissolved ferrous iron were conducted to evaluate the importance of mechanisms involving dissolved iron versus mechanisms involving the pyrite surface. In these experiments, ferrous and ferric iron concentrations were measured at the beginning and end of experiments with RNA. In one experiment, EDTA was added to an experiment with dissolved ferrous iron. This experiment was conducted to evaluate the role of EDTA on the rate of RNA degradation. To provide the basis for a comparison to the degradation rate in the presence of pyrite, dissolved iron concentrations in an experiment where RNA was added to a pyrite suspension was also measured at the start and end of the experiment. These control experiments were conducted in the presence of molecular oxygen.

## Materials and methods

### Pyrite sample preparation

All experiments were performed at room temperature (25 ± 2°C) with natural pyrite (Huanzala, Peru) obtained from Wards. All pyrite used in this study was crushed at the same time. After crushing it was washed with acid to remove surface oxides using a protocol described in earlier work [4]. The size fraction used in these experiments was between 10 to 90 μm with a five-point N_2 _adsorption BET surface area of roughly 1.25 m^2^/g. This BET surface area is an approximation due to the inherent variations for low surface area measurements [38]. Anaerobic experiments were performed in a glove box (Coy Laboratory Products) with Pd catalyst and O_2 _meter in the presence of nitrogen/hydrogen (95%/5%) gas. The concentration of molecular oxygen in N_2_-purged solutions was determined using a polarographic O_2 _probe (Orion).

### Hydrogen peroxide quantification

The H_2_O_2 _quantification technique for iron-containing solutions is described in detail elsewhere [39]. In brief, H_2_O_2 _oxidizes leuco crystal violet (LCV) (Spectrum) in the presence of the enzyme horseradish peroxidase (HRP type II) (Sigma) forming a crystal violet cation, which has an absorbance maximum around 590 nm [40]. Calibration curves were used to compensate for pH, ferrous iron, and EDTA. Varying amounts of pyrite were mixed with water in the absence or presence of 1 mM EDTA and immediately filtered (Millipore 0.45 μm). Catalase [Sigma (4 to 60) × 10^4 ^units/mg bovine liver] selectively degrades H_2_O_2 _and was used to verify that LCV oxidation was due solely from H_2_O_2_. Reagents were added to the aqueous filtrate in the following order with final concentrations: 100 mM KH_2_P_4 _pH buffer, 41 μM LCV (dissolved with HCl), and 1 μg HRP.

### ESR spin-trapping

The spin trap 5, 5-dimethyl, 1-pyrroline *N*-oxide (DMPO) was purified with activated carbon (Fisher). All spin trap experiments were conducted with a Bruker EMX ESR equipped with an Aqua-X liquid cell. Pyrite samples were exposed to DMPO solutions (for about 15 seconds), immediately filtered, injected into the liquid cell and immediately analyzed at room temperature (25 ± 2°C) in the presence of O_2_. The spectrometer settings are as follows: magnetic field of 3470 ± 100 G, microwave power of 20 mW, modulation frequency of 100 kHz and amplitude of 1 G, receiver gain of 2 × 10^5^, time constant of 0.64 sec, and scan time of 2 min 47 sec. Reagents and final concentrations during analyses: DMPO (Sigma) 100 mM, H_2_O_2 _120 μM, ferrous ammonium sulfate 25 μM, EtOH 30%, iron (III) sulfate pentahydrate 100 μM, pyrite 25 mg in 2 mL of solution.

### Nucleic acid quantification and visualization

Yeast RNA (Spectrum) was mixed with water, followed by centrifugation (4°C, 4500 × *g*, 5 min) and filtration (0.45 μm) to remove impurities. Experiments with pyrite were performed in 50-mL centrifuge tubes. 10 g/L pyrite was added to a 1.5 mg/L RNA solution, stirred and centrifuged (4°C, 4500 × *g*, 5 min) before sampling. RNA was quantified by using a molecular probe specifically designed for RNA (RiboGreen from Invitrogen). RiboGreen was added to solution aliquots following the manufacturers' protocol. Fluorescence measurements were conducted using a Picofluor instrument (Turner designs; excitation: 475 ± 15 nm, emission: 515 ± 10 nm). With this protocol it is possible to measure RNA strands with less than 100 bases [41]. When the initial RNA concentration had dropped to less than 98% (i.e., fragments were too small to interact with the probe), the RNA solution was replaced by a fresh solution. To avoid any exposure of the slurry to air, the vials were tightly closed in the anaerobic glove box, then centrifuged outside the glovebox. After this step, the vials were immediately placed back into the glove box, the supernatant decanted, and new N_2_-purged RNA solution added. After replacing the RNA solution, the periodic sampling and analysis protocol was resumed. In addition to using a molecular probe to quantify RNA, we also determined the RNA concentration using UV-Vis spectroscopy. Wavelength scans from 240 nm to 294 nm were performed on a Hach DR4000 spectrometer. Nucleic acid bases have an absorbance maximum at 260 nm. Ferrous iron dissolved from the pyrite surface absorbs light in this region with more light absorption as the wavelength decreases. A similar background absorbance occurs with RNA solutions. In order to simplify interpretation of the results, this sloped background absorbance has been subtracted from all of the curves.

To corroborate the studies using the RNA molecular probe or UV-Vis spectroscopy, we also conducted experiments with human ribosomal RNA and circular plasmid DNA. The experiments with human ribosomal RNA were performed by vortexing 60 g/L pyrite and 100 mg/L human ribosomal RNA extracted from HeLa cells (carcinoma cell line). The experiments were conducted in 2-mL centrifuge tubes. After periodic centrifugation and sampling, agarose gel electrophoresis was performed on the aqueous phase. Except for centrifugation and analysis, the experiment was performed in an anaerobic glove box. Using the same protocol, an experiment was carried out with circular plasmid DNA [5.5 kb in size pCDNA3 (Invitrogen) propagated in E.coli DH5-alpha and purified by HiSpeed Plasmid Maxi Kit (Qiagen)]. Unlike the experiment with human ribosomal RNA, the experiment with circular plasmid DNA was conducted in the presence of dissolved molecular oxygen.

### Dissolved iron analysis

Ferrous and ferric iron were quantified using HACH UV-Vis spectroscopic methods using 1, 10 phenanthroline and ferrozine, respectively. All analyses were conducted on filtered samples (0.45 μm pore size).

## Results

Results indicate that in an aqueous suspension, pyrite induces the spontaneous formation of H_2_O_2 _and ^•^OH. Nucleic acids added to pyrite suspensions degrade rapidly. Experiments with EDTA added to the pyrite suspensions demonstrate that the addition of this ligand stabilizes H_2_O_2 _and prevents ^•^OH formation and nucleic acid degradation.

### Pyrite-induced formation of hydrogen peroxide and hydroxyl radicals

Pyrite/aqueous suspensions spontaneously generate H_2_O_2_. In these experiments, pyrite was mixed with water containing EDTA and immediately filtered. Due to reaction of unchelated Fe(II) with H_2_O_2 _or to reaction of chelated Fe(II) with H_2_O_2_, increasing the reaction time reduces the concentration of H_2_O_2 _(Table 1). As seen in Table 1, the H_2_O_2 _concentrations increase proportional to particle loading. Catalase enzymatically degrades H_2_O_2 _to form O_2 _and H_2_O without the formation of radicals. When catalase is added to the pyrite suspension before analysis, H_2_O_2 _is not observed, which is consistent with H_2_O_2 _being the sole LCV oxidizer. Similar experiments performed in absence of O_2 _showed no detectible H_2_O_2 _(the detection limit for the LCV technique is about 0.5 μM; we are currently working on developing a protocol that will allow detection of H_2_O_2 _at nanomolar levels). In the presence of O_2_, H_2_O_2 _is only detected when the iron chelator, EDTA is added. In the absence of EDTA, H_2_O_2 _is not observed. The lack of H_2_O_2 _when iron is not chelated with EDTA suggests that most H_2_O_2 _formed in pyrite suspensions is rapidly transformed to ^•^OH. This rapid loss keeps the level of H_2_O_2 _below the detection limit of the LCV method. A second set of experiments to evaluate the stability of H_2_O_2 _in the presence of ferrous iron and ferrous iron with EDTA is presented in the bottom half of Table 1. When 10 μM Fe(II) is added to a 10 μM H_2_O_2 _solution, the H_2_O_2 _concentration is reduced to 4.2 μM. However, when 10 mM EDTA is added, H_2_O_2 _is stabilized.

**Table 1:** H_2_O_2 _Measurements*

**Pyrite loading (g/L)^a^**	**H_2_O_2_(μM)**
20 g/L with 1 mM EDTA	2.19
40 g/L with 1 mM EDTA	6.54
80 g/L with 10 mM EDTA	11.7
160 g/L with 10 mM EDTA	25.6
no EDTA added to any above	not detected
with EDTA and addition of catalase^b^	not detected

**Solution composition**	**H_2_O_2_(μM)**

10 μM H_2_O_2 _solution no pyrite	10
with 10 μM Fe(II)	4.2
with 1 mM EDTA	10.1
with Fe(II) and EDTA	10.1

*Experiments were performed in the presence of oxygen. ^a^Pyrite of varying particle loadings were suspended in EDTA, quickly filtered and analysed.

^b^127,000 units catalase/4 mL.

**Figure 1:**
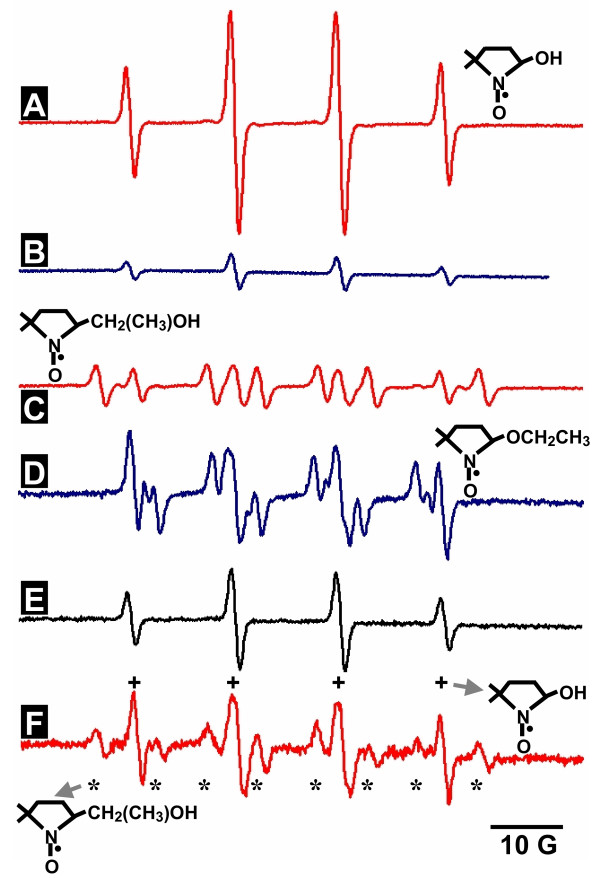
**ESR spin-trapping with 100 mM DMPO**. The structure of DMPO adducts are drawn on the figure. Fenton generated (125 μM H_2_O_2 _& 25 μM Fe^2+^) ^•^OH reacts with DMPO to form the DMPO-OH radical adduct (A). In the absence of ^•^OH, ferric iron (100 μM) also produces the DMPO-OH radical adduct (B). Reaction of ^•^OH with 30% ethanol results in the DMPO-CH(CH_3_)OH radical adduct (C). In the absence of ^•^OH, but presence of 100 μM ferric iron and ethanol, DMPO-OCH_2_CH_3 _results (D). Pyrite (12.5 g/L) mixed with DMPO (without addition of H_2_O_2_) shows formation of DMPO-OH (E). The addition of ethanol reveals peak contributions from the DMPO-OH (marked with plus signs) and ^•^OH-dependant DMPO-CH(CH_3_)OH radical adducts (marked with asterisks) (F). The experiment was conducted in the presence of O_2_. The raw ESR data are available as an additional file, Cohn_Fig 1 ESR data.XLS.

To evaluate whether pyrite generates ^•^OH when it is suspended in water, experiments were performed using ESR spin-trapping in the presence of dissolved molecular oxygen. ^•^OH reacts with the spin-trap DMPO forming the DMPO-OH radical adduct, which displays a four-line spectrum (Fig. 1A). In the absence of ^•^OH, the DMPO-OH adduct can also be formed via the interaction of the spin trap with ferric iron (B). In the presence of ^•^OH, ethanol reacts with ^•^OH to form the DMPO-CH(CH_3_)OH adduct (C). If ethanol is added to a solution of DMPO and ferric iron (i.e., no ^•^OH present), ethanol adds to DMPO resulting in the DMPO-OCH_2_CH_3 _adduct (D). DMPO-OCH_2_CH_3 _and DMPO-CH(CH_3_)OH exhibit different spectra (compare C and D). When DMPO is in the presence of pyrite, DMPO-OH is formed (E). Pyrite slurries contain trace amounts of ferric iron, so an experiment with ethanol is necessary. With ethanol, two sets of peaks result, one from the DMPO-OH adduct and one from the DMPO-CH(CH_3_)OH adduct (F), confirming the presence of ^•^OH. While these experiments clearly show that pyrite generates ^•^OH in the presence of water, it is not possible to determine the absolute concentration of the radical using the protocol presented above.

### Pyrite-induced degradation of nucleic acids

**Figure 2:**
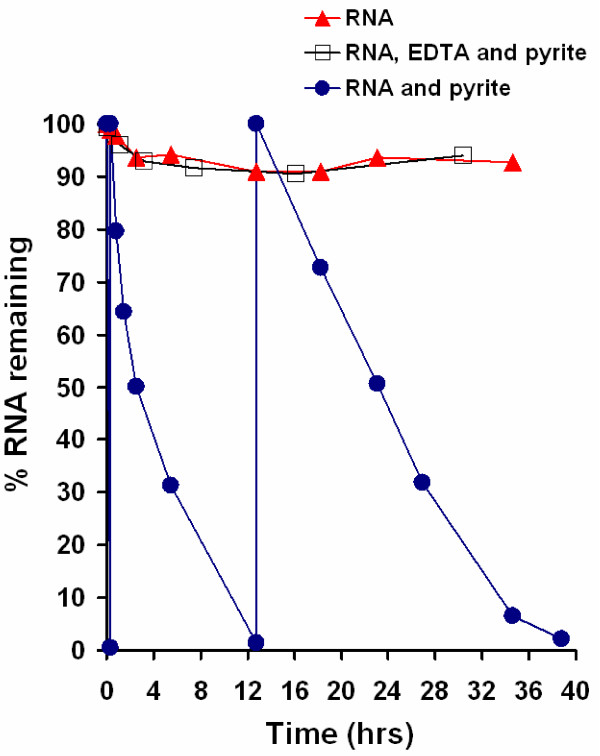
**Decomposition of 1.5 mg/L yeast RNA in the presence of 10 g/L pyrite**. RNA was exposed to pyrite in the absence of O_2_. Samples were taken after centrifuging the vials to prevent loss of pyrite. Upon 98% decomposition of RNA, the aqueous phase was removed and replaced with a fresh RNA solution. The experiment was continued beyond that shown. After seven days and three more replacements of the RNA solution, RNA continued to be degraded but at a slower rate. For comparison, an experiment with pyrite and RNA and the addition of 1 mM EDTA shows no loss in RNA.

**Figure 3:**
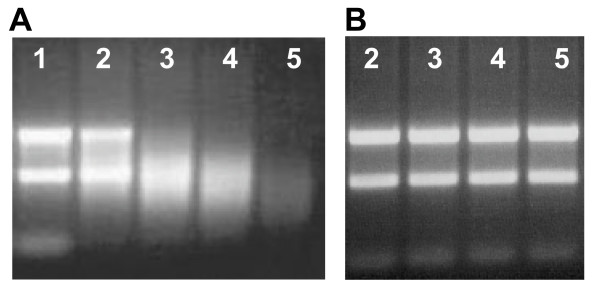
**Agarose gel electrophoresis of human ribosomal RNA (100 mg/L) vortexed with 60 g/L pyrite**. **A**: Periodic sampling of the pyrite/rRNA suspension (samples were centrifuged to remove pyrite particles). Lane 1 contained ribosomal RNA (the two bright fluorescent bands are due to 28S and 18S ribosomal moieties). Lanes 2–5 contain pyrite with samples taken after twenty seconds (2), thirty minutes (3), one hour (4), and two hours (5). **B**: Same procedure as A, however, in the presence of 10 mM EDTA (lane numbers correspond to the same exposure times as in **A**). The negatively-charged RNA samples were loaded at the top of the agarose gels. When a voltage is applied across the gel, the RNA strands travel downward with smaller strands moving faster than larger strands. The light intensity is indicative of quantity. The experiments were conducted in the absence of O_2_.

**Figure 4:**
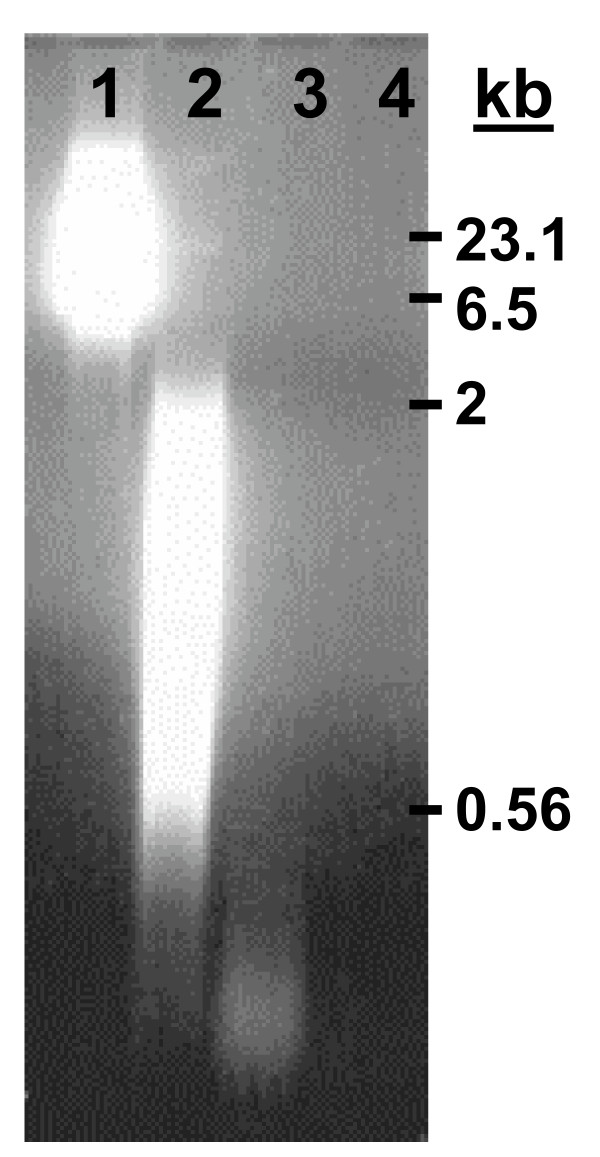
**Agarose gel electrophoresis of plasmid DNA (100 mg/L) with increasing amounts of pyrite**. DNA was exposed to pyrite for 4 hrs followed by centrifugation and sampling. The first lane is plasmid DNA, lane two: 6 g/L pyrite, lane three: 64 g/L pyrite, and lane four: 640 g/L pyrite. Positions of known strand lengths are given in kilo-bases on the right side of the figure. Similar to the RNA gel electrophoresis experiment, smaller DNA fragments move faster downward on the gel when a voltage is applied and the light intensity roughly correlates to quantity. The experiment was conducted in the presence of O_2_.

The reactivity of pyrite-generated ^•^OH toward nucleic acids was evaluated in the presence and absence of molecular oxygen. Figure 2 shows the pyrite-induced degradation of yeast RNA in the absence of molecular oxygen. The pyrite maintains this reactivity toward RNA even after the supernatant RNA solution is continuously refreshed. To investigate whether addition of an iron chelator will affect the degradation of RNA, EDTA was added. In the presence of EDTA, RNA remained stable, indicating that iron is necessary for the RNA-degradation mechanism. The source of iron is from the pyrite surface, but the mechanism may involve surface-bound iron or dissolved iron. The results are corroborated by those obtained with much larger RNA (i.e., ribosomal RNA) and analyzed using gel electrophoresis. Gel electrophoresis of human ribosomal RNA exposed to pyrite shows a decrease in the RNA fragment size and quantity over time in the absence of dissolved oxygen (Fig. 3a). A similar stabilization of RNA with EDTA (Fig. 3b) is seen here compared to the experiment with yeast RNA. Experiments were also carried out to address the effects of pyrite on DNA but with a different kind of experiment. Here, DNA was exposed to varying amounts of pyrite in the presence of molecular oxygen and samples were taken four hours after incubation. Results show greater DNA degradation with increasing amounts of pyrite (Fig. 4).

**Figure 5:**
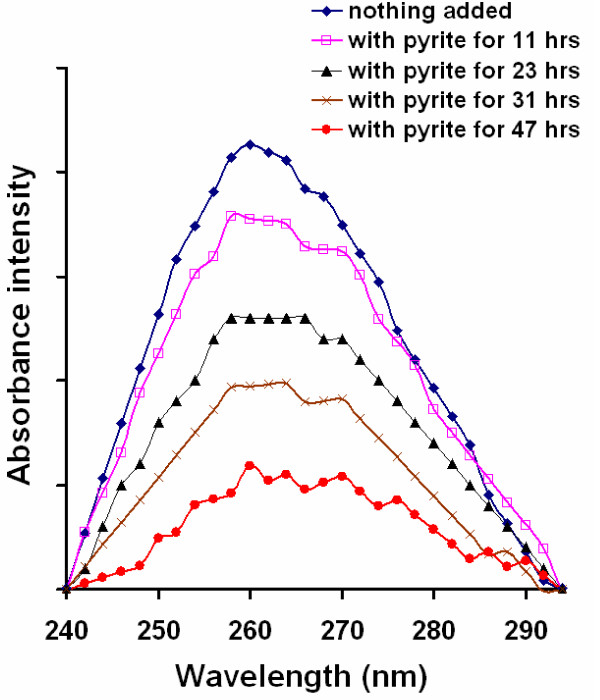
**UV-Vis wavelength scans of filtered yeast RNA/pyrite suspensions taken as a function of time**. 1.5 mg/L yeast RNA was exposed to pyrite (1 g/L) followed by periodic sampling. Wavelength scans were taken of the filtered samples. The loss of absorbance is putatively due to degradation of the nucleic acid bases. Background absorbance not associated with the absorbance peak at 260 nm (i.e., Fe(II) dissolved from pyrite or increasing absorbance at lower wavelengths due to RNA) has been subtracted from all of the curves. The raw UV-vis data and background corrected data are available as an additional file, Cohn_Fig 5 UV.Vis.XLS.

Collectively, the experiments show that the presence of pyrite leads to a decrease in nucleic acid strand length and a loss in fluorescence intensity. The fate of the nucleic acid bases was also investigated with UV spectroscopy. Samples of the yeast RNA samples exposed to pyrite were filtered to remove pyrite particles and wavelength scans were taken. The data shows a continual decrease in the absorbance centered around 260 nm when RNA is exposed to pyrite, which is consistent with a transformation of the bases that leads to a loss of their absorptivity at this wavelength (Fig. 5). In principle, the decrease of absorbance by itself could be due to simple removal of RNA from solution by adsorption. However, while simple sorption can explain a loss of fluorescence intensity in the gels, it cannot explain the shortening of the strands observed in these experiments.

### Control experiments

**Figure 6:**
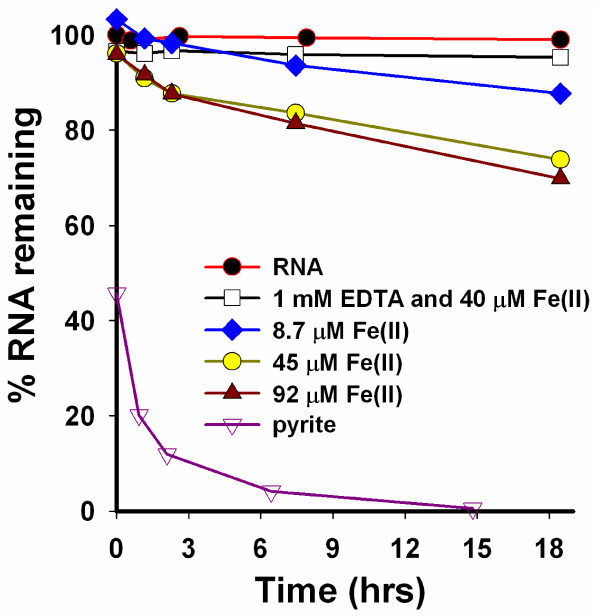
**Control experiments with dissolved ferrous iron**. 1.5 mg/L yeast RNA was exposed to different ferrous iron concentrations and an experiment with the addition of 1 mM EDTA. An experiment with 1 g/L pyrite is shown for comparison. The experiment was conducted in the presence of O_2_.

The mechanisms whereby pyrite generates ^•^OH, which leads to RNA degradation, may be a combination of solution reactions [e.g., Haber Weiss (eqs 1 and 2) and Fenton (eq. 7)] and surface-mediated reactions (eq. 3 and 4). Experiments, in the absence of pyrite, were performed to determine the rate of RNA degradation as a function of dissolved ferrous iron concentration in the presence and absence of EDTA in the presence of molecular oxygen (Fig. 6). For comparison, the results of an experiment with pyrite were also included in this figure. The iron concentrations at the start and the end of these experiments are summarized in Table 2. From these experiments, it is apparent that increasing solution concentrations of ferrous iron lead to higher rates of RNA degradation. However, the dissolved ferrous iron concentrations in these homogeneous control experiments are significantly higher than for the pyrite experiment shown in Figure 6, see Table 2. Iron concentrations decrease in experiments where iron was added to the beginning of the experiment, but increase when pyrite is added to an RNA solution. These changes in dissolved iron concentrations are due to precipitation of the ferrous iron that was added to RNA and dissolution of iron from the pyrite surface, respectively. The experiment with RNA exposed to ferrous iron and EDTA shows no RNA degradation.

**Table 2:** Dissolved Iron Concentrations (μM)*

	0 hours		16 hours	
Sample^a^	Fe(II)	Fe(III)	Fe(II)	Fe(III)
8.7 μM Fe(II)	8.7	0.5	5.3	1.6
45 μM Fe(II)	45	2.2	38	1.4
92 μM Fe(II)	92	3.5	78	0.3
pyrite	1.6	2.0	4.8	0.4

*Samples were taken from the RNA-containing vials, filtered and analyzed for ferrous iron and total iron at the beginning of the experiment and after 16 hours. ^a^Samples were taken from the experiment shown in Figure 6 (RNA mixed with either ferrous iron at varying concentrations or pyrite).

## Discussion

The results of the ESR spin trapping experiments show conclusively that ^•^OH is formed spontaneously in pyrite slurries in the presence of molecular oxygen. Complementary experiments with nucleic acids show a rapid strand shortening with loss of absorbance at 260 nm (Fig. 5), which is indicative of a reaction involving the bases in the nucleic acids. In earlier work [4,39], we showed that the rate of RNA degradation and the formation of H_2_O_2 _is proportional to the pyrite surface loading. Given that nucleic acids are rapidly decomposed by ^•^OH but stable in dilute H_2_O_2_, the results indicate that pyrite promotes the degradation of nucleic acids via a mechanism that converts H_2_O_2 _to ^•^OH. Exposure to pyrite leads to cleavage of the nucleic acid strands to a size that is eventually not detectable with RiboGreen (yeast RNA experiments Fig. 2) and too small to be retained on the agarose gels (human ribosomal RNA, Fig. 3, and circular plasmid DNA experiments, Fig. 4).

The mechanism for the H_2_O_2_-to-^•^OH conversion is not clear. The control experiments presented in Figure 6 show clearly that the presence of the pyrite surface promotes the degradation of nucleic acids and, by extension, the formation of ^•^OH. Experiments with dissolved iron at concentrations well above those in pyrite slurries show a much slower rate of RNA degradation. This suggests that a reaction mediated by the pyrite surface promotes the degradation. The mechanism whereby dissolved ferrous iron degrades RNA may be due to Haber-Weiss and Fenton chemistry (eqs 1, 2 and 7). The finding that EDTA inhibits the decomposition of the nucleic acid, but does not prevent the formation of H_2_O_2_, suggests that the H_2_O_2_-to-^•^OH conversion takes place primarily in solution. The rationale for this notion is that EDTA forms strong complexes with dissolved iron, which, under the conditions of these experiments, prevents the Fenton reaction from taking place (see control experiment with Fe(II) and EDTA in Fig. 6). The finding that H_2_O_2 _is formed in pyrite suspensions in the presence of EDTA and molecular oxygen precludes a strong interaction of the ligand with the pyrite surface. Electrochemical studies [42] as well as model calculations show that the transfer of electrons from the pyrite surface to adsorbed molecular oxygen takes place at iron sites on the surface [43]. If EDTA were to adsorb strongly onto the pyrite surface, the H_2_O_2 _formation via reactions 3 and 4 are expected to be inhibited. Hence, we speculate that pyrite promotes the degradation of nucleic acids and, by extension, the formation of ^•^OH by forming H_2_O_2 _at its surface which then can react with dissolved ferrous iron to form ^•^OH.

EDTA chelation may also prevent iron from binding with the RNA. Given that ^•^OH reacts almost instantaneously after it is formed, it has been suggested that sorption of iron to the nucleic acid strand is a prerequisite for its cleavage [28,29]. This is because low concentrations of ^•^OH formed in solution may react before reaching an RNA strand. However, reaction of iron bound to the phosphate moieties of RNA with H_2_O_2 _may cause a strand cut at that location. By chelating dissolved iron, this site-specific mechanism may be inhibited.

Even though H_2_O_2 _is not detected in pyrite slurries in the absence of molecular oxygen, experiments with yeast RNA as well as human ribosomal RNA conducted in an anaerobic glove box show nucleic acid degradation. In earlier work we suggested that a reaction between water and defects on the pyrite surface can produce H_2_O_2_. Once H_2_O_2 _is produced via this pathway it is likely to be converted to OH. The levels of H_2_O_2 _formed via this defect-driven mechanism are much lower than in the presence of molecular oxygen. The low steady-state concentration of H_2_O_2 _and the limitations of the H_2_O_2_-detection technique used here may explain why H_2_O_2 _was not detected in the absence of O_2_. The result of experiments with yeast RNA in which the RNA solution was replaced periodically indicate that this anaerobic mechanism is not easily exhausted. If the process was limited to surface defects, one would expect that the degradation of RNA would cease after one or two cycles of fluid replacement. By contrast, the reactivity is maintained over as many as five cycles (Figure 2), although the rate of the reaction decreases. We hypothesize that the semi-conducting properties of pyrite allow defects within the bulk of the pyrite crystals to effectively migrate to the surface (note that a ferric iron in the pyrite structure is electronically equivalent to an electron hole [34], which can migrate through the conduction band to the surface [44]). Experiments to evaluate this hypothesis are underway. In addition, experiments are underway to evaluate how aging of the pyrite after crushing will influence its reactivity.

Hydroxyl radicals react non-specifically with most organic molecules. Hence, the formation of OH in pyrite slurries, as demonstrated in this study, can lead to pyrite-induced transformation of organic molecules in any environment where pyrite reacts with water. This includes environments encountered in human lungs where pyrite may be present upon inhalation of coal or mine dust. In a separate study we will report on the formation of OH in coal as a function of pyrite content. The non-specific reactivity of OH toward organic molecules may also be exploited to degrade organic pollutants. *In situ*, Fenton-based chemical oxidation techniques, in which dilute H_2_O_2 _is injected into aquifers, have been applied to degrade organic solvents and other persistent organic compounds [12,13,15-17,19,31,45]. In these systems, iron (oxy)hydroxides or oxides are providing the iron to drive the Fenton reaction (eq. 7). The results of this study suggest that pyrite could also be effective as a mineral Fenton catalyst. Its widespread occurrence in reduced sediments [46,47] opens up the possibility to apply Fenton-based chemical oxidation techniques to reduced sediments. In addition, it may be possible to use injection of air to drive *in situ *chemical oxidation when pyrite is present in the subsurface. Finally, pyrite – a common mining waste product – may find use in *ex situ *engineered Fenton-based systems designed to treat water polluted with organic solvents. Although the use of pyrite in these systems may cause a secondary problem of acid production, remediation of acid may be more straightforward than degradation of an organic solvent.

## Conclusion

On the basis of a combination of ESR spin-trapping experiments and radical scavenging experiments with nucleic acids, we conclude that ^•^OH is formed spontaneously when pyrite is dispersed in water. The formation of ^•^OH, but not the formation of H_2_O_2_, is inhibited when EDTA is added to the pyrite suspensions, which suggests that the H_2_O_2_-to-^•^OH conversion takes place in solution. However, more research is needed to resolve the mechanism or mechanisms.

## Competing interests

The author(s) declare that they have no competing interests.

## Supplementary Material

Additional File 2 UV-Vis absorbance data used in Figure 5 in EXCEL spreadsheet format. Click here for file

Additional File 1 ESR data using in Figure 1 in EXCEL spreadsheet format. Click here for file
